# Prevalence and associated risk factors of low back pain among users of a primary health care clinic serving semi-urban and rural settlements in KwaZulu-Natal, South Africa

**DOI:** 10.4314/ahs.v22i2.68

**Published:** 2022-06

**Authors:** Khanyisile Khumalo, Firoza Haffejee

**Affiliations:** 1 Department of Chiropractic and Somatology, Durban University of Technology, Durban, South Africa; 2 Department of Basic Medical Sciences, Durban University of Technology, Durban, South Africa

**Keywords:** Low back pain, musculo-skeletal disorder, South Africa

## Abstract

**Background:**

Low back pain (LBP) is one of the most frequent musculoskeletal conditions and a common work-related health problem. In South Africa, people from lower socio-economic strata are involved in physical labour and also have unequal access to health services. There is minimal data on the prevalence of LBP in these communities. This study determined the prevalence and associated risk factors of LBP among public sector health care users in a semi-urban/rural area of KwaZulu-Natal, South Africa.

**Methods:**

The study was conducted at a primary health care clinic in the Umdoni municipality, KwaZulu-Natal, South Africa. Convenience sequential sampling was used. An interviewer-administered questionnaire was utilized due to literacy constraints. Participants (n=400) answered the questionnaire in either English or isiZulu.

**Results:**

The lifetime prevalence of LBP was 79.3%. Female gender and lifting heavy objects were associated with LBP. The direct impact of LBP was faced in the work place resulting in absenteeism, often followed by unemployment.

**Conclusion:**

In this setting, where the prevalence of LBP was high, specialized treatments for LBP were not available at the primary health care facility. Incorporation of such treatments will be useful, for people in lower socio-economic strata, to overcome the burden of LBP.

## Introduction

All over the world, lower socio-economic status is associated with a greater burden of ill-health, often due to the multiple socio-economic deprivations within these communities.^1^ In South Africa, the disproportionate living and working conditions within communities has led to poverty and unequal access to social and health services among communities from different socio-economic strata.^2^

In addition, there is a fragmentation of health services in South Africa between the public and private sectors.^2^ It is estimated that only 30% of all South African doctors work in the public sector, despite it serving over 80% of the country's population.^3^ There is also a substantial difference in resource availability between public and private sectors. Furthermore, within the public sector itself, majority of resources and spending is allocated to academic and other tertiary level hospitals, with only a minority devoted to non-hospital primary care centres / clinics, particularly those in peri-urban areas.^2^ These clinics are to a large extent serviced by nurses, who are often overworked and hence unable to provide a personal service to patients.^4^ Communicable diseases like human immunodeficiency virus (HIV) and tuberculosis (TB) are treated at the primary care level, as are non-communicable diseases such as hypertension and diabetes.^4^ There are no specialist medical services. Although, allied health care services such as physiotherapy are offered, complementary alternative treatment, such as chiropractic treatment, for musculoskeletal disorders do not form part of the package of services at any level of care in the public sector of SA. Chiropractors offer manual treatments which are a more effective form of long-term treatment for low back pain (LBP) compared to drug therapy.^5^

Low back pain is one of the most frequent musculoskeletal conditions. According to Jin, et al.,^6^ in economically developed countries, LBP is one of the most common work-related health problems. In a study conducted to investigate the prevalence of occupational LBP for intensive labour, the lifetime prevalence ranged between 60–90% in countries worldwide.^7^ Research indicates that people involved in occupations that require awkward postures and physical labour are predisposed to LBP due to their tasks, which cause stress on the spine.^8^ For instance, LBP prevalence in nurses and welders is as high as 70.8%.^9^,^10^,^11^

Poor posture such as stooping, carrying of heavy loads, body vibration and repetitive tasks may predispose an individual to LBP.^8^,^12^–^15^ Carrying heavy loads repeatedly causes spine loading which in turn causes mechanical damage to tissues.^16^ Furthermore, vigorous repetitive loading can cause multiple micro-cracks in bones as well as injury to the vertebral discs, which then results in LBP.^17^ Therefore, atrophy in muscle, bone and cartilage may result in an inability of these structures to function under high loads such as direct impacts and falls.^17^

Low back pain causes a reduced quality of life and also places a burden on health care.^14^,^19^,^20^ The economic burden of LBP may be of particular concern in Africa since limited health funds are directed towards other epidemics such as TB, HIV and AIDS.^18^ In addition, individuals who reside in rural areas are generally poor, illiterate and have limited access to health care services, which may compromise their health status.^21^,^22^

In South Africa the lifetime prevalence of LBP varies from 48% to 78.2% in different sub-groups of the urban population.^23^,^24^,^25^ However, the prevalence of LBP in rural communities in the country has not been investigated. Since epidemiological studies are required to monitor the disease burden in any community and to implement control strategies, it is important to ascertain the prevalence of LBP in rural communities in South Africa. We therefore aimed to determine the prevalence and associated risk factors of LBP among primary health care clinic users in the Umdoni Municipality, which includes semi-urban towns and rural settlements, in the province of Kwa-Zulu Natal, South Africa.

## Methods

### Setting

This study was conducted in the Municipal Clinic situated in the town of Umzinto in the province of KwaZulu-Natal, South Africa. This clinic serves the population of the Umdoni Municipality, which is located 50 km south of Durban and covers an area of 251.53 square kilometres. 26,27,28 Several small semi-urban towns and many rural settlements, enclosed within farms and traditional authority land are located within this municipal area. The total population is 78 875, of which 76.72% are Black African, 8.50% White, 13.32 % Indian or Asian and 1.46% are made up of Coloureds and other population groups that were not specified.^26^–^30^ A minimum sample size of 384 was calculated based on the total population located within the area, a confidence interval of 95% and 5% margin of error.

### Questionnaire

The questionnaire was modified from a previously validated questionnaire by Docrat.^24^ The modification was necessary for suitability of some questions for this study population. The questionnaire comprised of sections on demographic information, daily activities, access to water, LBP prevalence and characteristics as well as health history. The prevalence of LBP was assessed using a question on whether they had LBP, with a yes/no option for the answer. All questions were close-ended with tick boxes provided for the answers. An expert group of five people verified content validity of the questionnaire. Following the expert group discussion, the questionnaire was piloted with five bilingual participants from the study area. This provided construct validity, which measures the accuracy of the answers to the questionnaire in relation to the reflection of the theoretical predictions of a certain construct.^31^

It also ensured that culturally sensitive, comprehensive and accurate information was captured thoroughly from the participants. Questionnaires and consent forms were provided in both English and isiZulu, which is the home language of the indigenous people from the research setting.

### Sample recruitment and data collection

The inclusion criteria for the study were as follows: residents of the Umdoni Municipality, 18 years and over, of any race or gender and fluent in either English or isiZulu. Anyone related to the researchers or who participated in the pilot study were excluded. A convenience sequential sampling method was utilized to recruit patients in the waiting room at the Umzinto Municipal Clinic. Convenience sequential sampling refers to choosing individuals, who are conveniently available and wiling to participate in the research.^32^ This allowed for sampling of a low socio-economic population in primaralily a rural and semi-rural area. The study was explained to small groups of patients in the waiting room of the clinic. Those who were interested in participating were read out the letter of information and subsequently required to sign a consent form in their language of choice, either English or isiZulu. Due to the limited degree of literacy of patients at the clinic, all participants were interviewed by the researcher, who then scribed the answers provided by the participants onto the questionnaire. To avoid bias, all questions were phrased exactly as they appeared on the questionnaire and the answers were scribed verbatim, without any prompting.

In addition to answering the questionnaire, the weight and height measurements of each participant was taken using a digital scale and stadiometer, respectively. These measurements were used to subsequently calculate the body mass index (BMI) as follows: BMI = weight /height^2^ (kg/m^2^). A normal BMI range is 18.5–24.9. 33

### Ethics

Ethical clearance was obtained from the Institutional Research Ethics Committee (IREC 120/16) prior to commencing the study. Gatekeeper permission was obtained from the Department of Health, KwaZulu Natal and Umdoni Municipality Primary Care Clinic Manager.

### Data analysis

The statistical program for the Social Sciences (SPSS) version 23 was utilized for data management and statistical analysis. Descriptive and inferential statistics were used in the analysis. Pearson's chi square tests and Fisher's exact test, as appropriate were utilized in order to determine the association between LBP and various factors.^34^ Odds ratios and 95% confidence intervals were calculated, where relevant. Where the bivariate p value was < 0.05, those variables were entered into a multiple logistic regression model using backward selection to eliminate non-significant predictors, until a final model was reached where only significant predictors remained.

## Results

### Demographics

A total of 400 participants with a median age of 38.4 years (range: 18–82), completed the questionnaire. Table 1 shows the full demographic characteristics of the participants, who were predominantly female (69.3%, n = 277) and of the Black African race (97.1%, n = 389). Most participants were either overweight or obese and the mean body mass index (BMI) was 27.6 ± 5.63 kg/cm^2^.

**Table 1:** Sociodemographic of study participants

Demographic factor	*n* (%)
**Gender**	
Females	277 (69.25)
Males	123 (30.8)
**Race**	
Black African	389 (97.1)
Other	11 (2.9)
**Relationship status**	
Single	218 (54.5)
Married	66 (16.5)
Cohabiting	62 (15.5)
**Level of education**	
No formal education	66 (16.5)
Primary school	99 (24.8)
Secondary school	
Currently attending/incomplete	151 (37.8)
Matriculated	55 (13.8)
Tertiary	
Certificates	7 (1.6)
Diplomas	22 (5.5)
**Employment**	
Unemployed	201 (50.3)
Part time employment	67 (16.8)
Full time employment	33 (8.3)
Self employed	11 (2.8)
Students	40 (10)
Retired	19 (4.8)
**Monthly income (South African Rands)**	
<1 500	276 (68.9)
1 500 – 3 000	78 (19.5)
>3 000	46 (11.6)
**Body Mass Index (BMI)**	
Underweight (<18.5)	13 (3.2)
Normal (18.5–24.9)	132 (33)
Overweight (25–29.9)	155 (38.8)
Obese (>30)	100 (25)

NB: Percentages do not always total 100% as some questions were not answered by all participants

Education levels were generally low and unemployment rates were high. Those who were employed were either domestic workers (3.3%, n = 13) or some other form of unskilled workers (5%, n = 20). Income levels were low, with the majority earning less than R1500 per month (equivalent to US$ 86).

Table 2 indicates the socio-demographic characteristics, related to water availability. Only 7% (n = 28) had access to piped water in their homes and hence the majority had to carry large volumes of water from communal taps or river sources, often at a distance of a few kilometers.

**Table 2:** Water conditions in communities

Water condition	*n* (%)
Water availability	
Access to piped water in home	28 (7)
Common taps in communities	194 (48.5)
Rivers	121 (30.3)
Volume of water carried (litres)	
0–15	7 (1.8)
16–20	234 (58.5)
21–25	73 (18.2)
More than 25	1 (0.3)
Distance travelled between water source and home (kilometres)	
0–2	79 (19.8)
3–5	147 (36.8)
6–8	82 (20.5)
More than 8	7 (1.7)
Weekly frequency of fetching water	
1–3 times	22 (5.5)
4–6 times	58 (14.5)
7–10 times	112 (28)
11–14 times	112 (28)
More than 14 times	11 (2.8)
Method of carrying water	
Carrying bucket on head	215 (53.8)
Carrying bucket by hand	43 (10.8)
Using a wheelbarrow	57 (14.2)

NB: Percentages do not always total 100% as some questions were not answered by all participants

More females (98%, n = 272) performed household chores than did males (78.8%, n = 97, p < 0.001). These chores included fetching firewood (p < 0.001), carrying large volumes of water (p = 0.001), washing clothes (p < 0.001) and cooking (p < 0.001).

### Prevalence and risk factors of low back pain

The lifetime prevalence of LBP was 79.3% (n = 317) and the point prevalence was 32.5% (n = 130). Characteristics related to LBP are indicated in Table 3, which shows that the frequency of occurrence was high, with over a third reporting weekly recurrence of LBP. More than two-thirds (70.5%) also reported that the pain was getting progressively worse with time.

**Table 3:** Low back pain (LBP) characteristics

	*n (%)*
**Prevalence**	
Lifetime prevalence	317 (79.3)
Point prevalence	130 (32.5)
**Age at first onset (years)**	
≤ 15	5 (1.25)
16–20	55(13.75)
21–30	108 (27)
31–40	57 (14.25)
41–50	47 (11.75)
51–60	36 (9)
61–70	10 (2.5)
**Frequency**	
Everyday	27 (6.8)
Once a week	45 (11.2)
2–3 times a week	26 (6.5)
Every second week	3 (0.8)
Once a month	15(3.9)
Occasionally	78 (19.3)
	206 (51.5)
**Pain progression**	
LBP is getting worse	91 (22.7)
LBP is decreasing	7 (1.8)
Constant	31 (7.7)

NB: Percentages do not always total 100% as some questions were not answered by all participants

Low back pain was more prevalent with increasing age (p = 0.028). Similarly, LBP increased in people with higher BMI (p < 0.001). More females (83.4%, n = 231) suffered from LBP than did males (69.9%, n = 86, p = 0.002). The prevalence of LBP was not correlated with other demographic factors.

Table 4 indicates the results of the bivariate analysis. The age categories were dichotomized to ≥40 years and under 40 years, prior to conducting the analysis. Female gender, over the age of 40 years, smoking, lifting heavy objects, carrying water, arthritis and tuberculosis were all associated with low back pain. After adjusting for all factors, the multivariate analysis indicates that female gender and lifting heavy objects were associated with LBP. No other factors were associated (Table 4).

**Table 4:** Bivariate and multivariate analysis for risks associated with low back pain

	Bivariate Analysis			Multivariate Analysis		

	Crude OR	95% CI	*p* value	Adjusted OR	95% CI	*p* value
Female	2.16	1.31–3.16	0.002	3.76	1.91–7.41	<0.001*
Age ≥ 40 years	4.16	2.25–7.69	<0.001	2.24	0.87–5.78	0.095
Smoking	2.12	1.00 –4.78	0.028	1.86	0.21–16.48	0.575
Prolonged standing	1.67	0.88 –3.18	0.075	0.99	0.32–3.10	0.996
Lifting heavy objects	6.01	2.13 – 16.98	<0.001	9.96	2.17–45.89	0.003*
Carrying water	1.60	1.24 –2.08	<0.001	1.53	0.78–50.16	0.208
Arthritis	6.72	1.74 – 18.77	<0.001	3.68	1.68–8.04	0.096
Tuberculosis	2.48	1.81 – 5.66	0.024	1.68	0.61–4.61	0.315

### Impact of low back pain

The majority of the participants reported that their LBP affected daily activities such as bending, dressing, driving, lifting objects, sitting, sleeping, standing and walking. The main difficulties experienced were bending (30.5%, n = 122) and lifting of heavy objects (18%, n = 72). Some participants (14.3%, n = 57) were bed-ridden due to LBP and consequently absent from work. Furthermore, some participants had to change their jobs (0.8%, n = 3) while others stated that they lost their jobs because of the LBP (6.3%, n = 25).

### Treatment

About 66.9% (n = 83) of the participants were on medication for LBP. More than three-quarter of whom (77%, n = 64) received their medication from the government clinic. Some sought treatment from traditional healers (19.2%, n = 16). Of the participants who were currently on LBP medication, 72.3% (n = 60) felt that the medication was not helping to ease the pain. Although physiotherapy is offered at the clinic, none of the participants received this treatment for LBP. The majority of the participants (99%, n = 396) were unaware of complementary alternate therapies, including chiropractic. This service is not offered in the area, both at the clinic or privately, and was therefore not sought by any of the participants.

## Discussion

This study ascertained the factors associated with LBP among semi-urban and rural South Africans attending a public health care facility. We report a lifetime prevalence of 79.3% among this population. Associated factors included female gender and lifting heavy objects.

Our findings are higher than those reported in developed countries.^35^,^36^ Furthermore, the prevalence is higher than that of city dwelling Black Africans (57.6%) in the same province and that of the White population (48%), who reside in urban areas of South Africa,^23^,^25^ indicating a disproportionate burden of LBP among different population groups in the country. Poverty was an overarching factor among this community of people who were either unemployed or with low monthly income. Many did not have tangible infrastructural amenities such as clean, piped water. Whilst multivariate associations did not show a direct causal association between this amenity and LBP, there is a direct association between lifting heavy objects and LBP. Participants indicated that they carried large volumes of water over a long distance. Many carried a heavy bucket of water, of up to 20 litres, on the head or by hand, as opposed to using a wheelbarrow or cart to transport the water. This affects the biomechanics of the spine leading to musculoskeletal dysfunction and early degeneration of the bones, joints and soft tissues.^37^,^38^ The increased load is usually conveyed to the lumbar region, with a resultant possible injury to the vertebrae of the low back.^15^ Furthermore, carrying heavy water on the head has deleterious effects on girls, whose physical immaturity can lead to spinal deformity, which may cause problems in future pregnancy and child birth.^39^

Other heavy objects that were carried, included firewood, indicating a lack of electricity in the homes. More women than men performed these chores, possibly contributing to the higher prevalence of LBP among women. This higher prevalence among women in a similar community in Lesotho, was also reported by Worku^22^, who alluded to women in African rural communities being expected to perform all the domestic work and to also be involved in intensive farming of crops for food.^22^ Other reports also indicate that in Africa, domestic load-carrying duty is seen as a low status activity and is usually performed by women and their children.^39^

Furthermore, low back pain was associated with BMI. Obesity affects the biomechanics of the spine, which may cause friction on the articulating surfaces, resulting in wear and tear of the joints with subsequent LBP.^40^

We report that LBP resulted in absenteeism from work, as participants were sometimes bedridden due to the severity of the pain. Indeed, some reported that they lost their employment due to this pain, resulting in a further economic burden to people from a low-income community. Low back pain is known to have a high economic burden due to lack of productivity as a result of work absenteeism.^14^

Participants in the current study were underprivileged and of low socio-economic status. Consequently, many did not seek medical assistance for LBP, which could have exacerbated the underlying condition. Limited access to health care services have been reported to compromise the health of individuals.^21^,^22^ Previous studies have indicated that a low level of education and low income are associated with LBP.^41^,^42^ A higher level of education and income may be important in providing knowledge of aspects related to LBP.^43^ Higher education would also result in better employment opportunities, involving fewer menial tasks and hence a decreased incidence of LBP. Hence lower socio-economic groups suffer numerous deprivations. This is across various countries, where the poor face many predisposing factors, recognized as social determinants of ill-health. They also cannot afford to seek care when ill.^44^

It is also noted that the primary health care clinics do not provide complementary alternate therapy, such as chiropractic, which is useful in the treatment of musculo-skeletal disorders. In fact, the participants were unaware of such therapy. Chiropractic procedures such as spinal manipulative therapy, which involves the application of a high-velocity, low-amplitude thrust to the spine, have been found to be beneficial in the long term treatment of LBP.^45^ Such manual treatments are reported to be more effective than drug therapy in the treatment and long term management of LBP.^5^ In addition, chiropractic treatment reduces chronic problems and decreases hospitalization and individuals who are treated for LBP by chiropractors return to work earlier.^46^ Acute and chronic bouts of LBP have received high success rates with chiropractic treatment. Our findings thus suggest a need for a chiropractic clinic in the area or the incorporation of chiropractic treatment in primary health care centres in South Africa. This will address some of the inequalities regarding access to healthcare among the lower socioeconomic strata within the country especially access to alternative complementary healthcare.

## Conclusion

There is a high prevalence of LBP among semi-urban and rural dwellers of KwaZulu-Natal, South Africa. Lifting of heavy objects was associated with LBP. The study findings suggest incorporating alternative complementary healthcare professionals in primary health care settings, as this will benefit patients. This in-turn will help alleviate the burden faced by the healthcare system where healthcare professionals from traditional and complementary backgrounds facilitate a holistic approach for patient-centered care. This will be particularly useful, for people in lower socio-economic strata, to overcome the burden of LBP without compromising their livelihoods.

## Limitations

The study findings derived from using convenience sampling may not be used as a generalization for other population areas due to the lack of basic facilities within this study area.

## Recommendations

There is a need to alleviate the burden of disease by decreasing the social and structural determinants of health. The provision of clean, piped water will not only prevent water-borne infections but also decrease non-communicable disease such as back pain within low socio-economic communities. The high prevalence of LBP within this community, also indicates the necessity of incorporating chiropractic treatment as an option in primary health care clinics in South Africa, as manual treatments offered by chiropractors are effective in the treatment of LBP. This will improve access to a full range of health services to communities with low levels of income.

## References

[1] Gwatkin DR (2000). Health inequalities and the health of the poor: What do we know? What can we do?. Bulletin of the World Health Organisation.

[2] Coovadia H, Jewkes R, Barron P, Sanders D, McIntyre D (2009). The health and health system of South Africa: historical roots of current public health challenges. The Lancet.

[3] Keeton C (2010). Bridging the Gap in South Africa. Bulletin of the World Health Organization.

[4] Maillacheruvu P, McDuff E (2014). South Africa's return to primary care: The struggles and strides of the primary health care system. The Journal of Global Health.

[5] Hill JC, Whitehurst DG, Lewis M, Bryan S, Dunn KM, Foster NE (2011). Comparison of stratified primary care management for low back pain with current best practice (STarT Back): a randomised controlled trial. The Lancet.

[6] Jin K, Sorock GS, Courtney TK (2004). Prevalence of low back pain in three occupational groups in Shanghai, People's Republic of China. Journal of safety research.

[7] Bell JA, Burnett A (2009). Exercise for the primary, secondary and tertiary prevention of low back pain in the workplace: a systematic review. Journal of occupational rehabilitation.

[8] Roffey DM, Wai EK, Bishop P, Kwon BK, Dagenais S (2010). Causal assessment of awkward occupational postures and low back pain: results of a systematic review. The Spine Journal.

[9] Jensen JN, Holtermann A, Clausen T, Mortensen OS, Carneiro IG, Andersen LL (2012). The greatest risk for low-back pain among newly educated female health care workers; body weight or physical work load?. BMC Musculoskeletal Disorders.

[10] Sikiru L, Hanifa S (2010). Prevalence and risk factors of low back pain among nurses in a typical Nigerian hospital. African health sciences.

[11] Sikiru L, Shmaila H (2009). Prevalence and risk factors of low back pain among nurses in Africa: Nigerian and Ethiopian specialized hospitals survey study. East African journal of public health.

[12] Macfarlane GJ, Jones GT, Hannaford PC (2006). Managing low back pain presenting to primary care: where do we go from here?. Pain.

[13] Wai EK, Roffey DM, Bishop P, Kwon BK, Dagenais S (2010). Causal assessment of occupational bending or twisting and low back pain: results of a systematic review. The Spine Journal.

[14] Wai EK, Roffey DM, Bishop P, Kwon BK, Dagenais S (2010). Causal assessment of occupational lifting and low back pain: results of a systematic review. The Spine Journal.

[15] Wai EK, Roffey DM, Bishop P, Kwon BK, Dagenais S (2010). Causal assessment of occupational carrying and low back pain: results of a systematic review. The Spine Journal.

[16] Riihimäki H (1991). Low-back pain, its origin and risk indicators. Scandinavian Journal of Work, Environment & Health.

[17] Dolan P, Adams MA (2001). Recent advances in lumbar spinal mechanics and their significance for modelling. Clinical Biomechanics.

[18] Louw QA, Morris LD, Grimmer-Somers K (2007). The prevalence of low back pain in Africa: a systematic review. BMC Musculoskeletal Disorders.

[19] Majid K, Truumees E (2008). Epidemiology and natural history of low back pain. Seminars in Spine Surgery.

[20] Maniadakis N, Gray A (2000). The economic burden of back pain in the UK. Pain.

[21] Galukande M, Muwazi S, Mugisa DB (2005). Aetiology of low back pain in Mulago Hospital, Uganda. African health sciences.

[22] Worku Z (2000). Prevalence of low-back pain in Lesotho mothers. Journal of Manipulative and Physiological Therapeutics.

[23] Van der Meulen AG (1997). An epidemiological investigation of low back pain in a formal Black South African township.

[24] Docrat A (1999). A comparison of the epidemiology of low back pain in Indians and Coloured communities in South Africa.

[25] Dyer BA (2012). An epidemiological investigation of low back pain in the white population of the greater eThekwini metropolitan area.

[26] (2005). Statistics South Africa. South African statistics 2004/05.

[27] (2011). Stats SA. Statistics South Africa. Formal census.

[28] Zissette S (2015). “If You Don't Take a Stand for Your Life, Who Will Help You?”: A Qualitative Study of Men's Engagement with HIV/AIDS Care in Rural KwaZulu-Natal, South Africa.

[29] Lehohla P (2005). South African Statistics, 2004/05.

[30] Lehohla P (2012). Census 2011 Municipal report KwaZulu-Natal.

[31] Black HC, Garner BA, McDaniel BR (1999). Black's law dictionary.

[32] Collins KM, Onwuegbuzie AJ, Jiao QG (2006). Prevalence of mixed-methods sampling designs in social science research. Evaluation & Research in Education.

[33] Flegal KM, Carroll MD, Kit BK, Ogden CL (2012). Prevalence of obesity and trends in the distribution of body mass index among US adults, 1999–2010. JAMA.

[34] Freburger JK, Holmes GM, Agans RP, Jackman AM, Darter JD, Wallace AS (2009). The rising prevalence of chronic low back pain. Archives of Internal Medicine.

[35] Vieira ER, Kumar S, Coury HJ, Narayan Y (2006). Low back problems and possible improvements in nursing jobs. Journal of Advanced Nursing.

[36] Walker BF, Muller R, Grant WD (2004). Low back pain in Australian adults. Prevalence and associated disability. Journal of manipulative and physiological therapeutics.

[37] Geere J-AL, Hunter PR, Jagals P (2010). Domestic water carrying and its implications for health: a review and mixed methods pilot study in Limpopo Province, South Africa. Environmental Health.

[38] Porter G, Hampshire K, Dunn C, Hall R, Levesley M, Burton K (2013). Health impacts of pedestrian head-loading: A review of the evidence with particular reference to women and children in sub-Saharan Africa. Social Science & Medicine.

[39] Porter G, Hampshire K, Abane A, Munthali A, Robson E, Mashiri M (2012). Child porterage and Africa's transport gap: Evidence from Ghana, Malawi and South Africa. World Development.

[40] Leboeuf-Yde C, Wedderkopp N, Andersen LB, Froberg K, Hansen HS (2002). Back pain reporting in children and adolescents: the impact of parents' educational level. Journal of manipulative and physiological therapeutics.

[41] Deyo RA, Mirza SK, Martin BI (2006). Back pain prevalence and visit rates: estimates from US national surveys, 2002. Spine.

[42] Hoy D, Brooks P, Blyth F, Buchbinder R (2010). The epidemiology of low back pain. Best practice & research. Clinical Rheumatology.

[43] Bener A, Dafeeah EE, Alnaqbi K (2014). Prevalence and correlates of low back pain in primary care: what are the contributing factors in a rapidly developing country. Asian spine journal.

[44] Wagstaff A (2002). Poverty and health sector inequalities. Bulletin of the World Health Organization.

[45] Coulter ID, Hurwitz EL, Adams AH, Genovese BJ, Hays R, Shekelle PG (2002). Patients Using Chiropractors in North America: Who Are They, and Why Are They in Chiropractic Care?. Spine.

[46] Dagenais S, Tricco AC, Haldeman S (2010). Synthesis of recommendations for the assessment and management of low back pain from recent clinical practice guidelines. The Spine Journal.

